# Signal Processing and Target Fusion Detection via Dual Platform Radar Cooperative Illumination

**DOI:** 10.3390/s19245341

**Published:** 2019-12-04

**Authors:** HuiJuan Wang, ZiYue Tang, YuanQing Zhao, YiChang Chen, ZhenBo Zhu, YuanPeng Zhang

**Affiliations:** 1 Air Force Early Warning Academy, Wuhan 430019, China; wy_wanghuijuan@163.com (H.W.); tang_zi_yue@163.com (Z.T.); zzbradar@126.com (Z.Z.); zhangyuanpeng312@163.com (Y.Z.); 2 The 93552 Troop of Chinese People’s Liberation Army, Shijiazhuang 050081, China; zyq_zhao@163.com

**Keywords:** cooperative illumination, cooperative detection, signal processing, target fusion detection

## Abstract

A modified signal processing and target fusion detection method based on the dual platform cooperative detection model is proposed in this paper. In this model, a single transmitter and dual receiver radar system is adopted, which can form a single radar and bistatic radar system, respectively. Clutter suppression is achieved by an adaptive moving target indicator (AMTI). By combining the AMTI technology and the traditional radar signal processing technology (i.e., pulse compression and coherent accumulation processing), the SNR is improved, and false targets generated by direct wave are suppressed. The decision matrix is obtained by cell averaging constant false alarm (CA-CFAR) and order statistics constant false alarm (OS-CFAR) processing. Then, the echo signals processed in the two receivers are fused by the AND-like fusion rule and OR-like fusion rule, and the detection probability after fusion detection in different cases is analyzed. Finally, the performance of the proposed method is quantitatively analyzed. Experimental results based on simulated data demonstrate that: (1) The bistatic radar system with a split transceiver has a larger detection distance than the single radar system, but the influence of clutter is greater; (2) the direct wave can be eliminated effectively, and no false target can be formed after suppression; (3) the detection probability of the bistatic radar system with split transceivers is higher than that of the single radar system; and (4) the detection probability of signal fusion detection based on two receivers is higher than that of the bistatic radar system and single radar system.

## 1. Introduction

With the increasing complexity of the battlefield electromagnetic environment, the single airborne early warning (AEW) radar is facing increasingly more challenges [1,2,3]. On the one hand, limited by the detection range, the single AEW is easy to be detected by the enemy; on the other hand, the maneuverability of AEW is limited, and the battlefield survivability is weak. An idea to solve this problem is to cooperate with a multi-platform radar system. Specifically, the cooperative detection mode of the AEW and unmanned aerial vehicle (UAV) has attracted extensive research [4,5,6]. In general, the UAV is in front and receives signals passively while the AEW is located in the safe rear and receives signals actively. In this model, the detection range can be expanded, and the problems of a huge radar blind area and radar detection efficiency caused by a large time-bandwidth signal can be improved to a certain extent [7,8,9,10]. Moreover, the separation of receiving and transmitting of the system is helpful to improve the detection ability of the stealth target and the anti-jamming ability [11,12,13,14]. In a summary, the cooperative detection mode of AEW and UAV is of great research significance.

Clutter and direct-wave interference are the main challenges for multi-platform cooperative detection. Recently, many researchers have carried out relevant research on airborne cooperative detection, and lots of cooperative detection methods have been proposed [15,16,17,18,19,20]. In [15], Wu studied the clutter modeling of an airborne bistatic radar based on a geometric model. Wei [16] studied the range-dependent compensation method for airborne bistatic radar clutter, in which the projection point of the receiver on the horizontal plane was represented as the origin and the projection of the baseline on the horizontal plane as the *Y* or *X* axis. Based on models of [15,16], Yang [17] analyzed the non-stationary characteristics of the clutter spectrum with range space variation and the serious aliasing characteristics of the clutter caused by range and Doppler ambiguity. A knowledge-assisted space-time adaptive processing method was proposed, which greatly improves the performance of clutter suppression of the high-speed airborne radar. In the aspect of suppressing the direct wave, Chen [18] proposed a method of direct wave suppression based on subspace projection, where an orthogonal projection matrix of a direct wave subspace was constructed using the equivalent signal of the receiving end, and the received signal was projected to suppress direct wave interference. The authors of [19] investigated a bistatic sonar for underwater target detection, and provided a deep zero trap to suppress direct wave signals in the direct wave direction through the beam zero suppression algorithm. In [20], for the two-channel passive radar system, the least square method of vector space was used to suppress the direct wave signals in the target channel, although it could not completely suppress the direct wave signals, and some of them were left. The authors of [21,22,23] mainly described the algorithm of time-reversal imaging, which was used to analyze the process of active detection. The authors of [24] focused on the classic problem of testing samples drawn from independent Bernoulli probability mass functions, when the success probability under the alternative hypothesis was unknown. Most of the above literature conducted studies on clutter suppression or direct wave suppression or fusion detection separately, mainly considering the problem of improving SNR. Moreover, the overall research background was inconsistent and there were many constraints. However, there were few joint studies on direct wave suppression under the clutter condition, and few studies on the influence of direct wave suppression on target fusion detection or positioning.

In this paper, we studied the signal processing and target fusion detection of an airborne cooperative detection system under the clutter condition. First, we constructed a dual platform cooperative detection system geometric model of the application scene, which uses AMTI to suppress clutter. Then, the AMTI, pulse compression, and phase-coherent accumulation were used to suppress the direct wave to suppress the influence of the false target generated by the direct wave during target detection. Finally, the echo received by the two receivers was used for fusion detection to improve the detection performance of the target. The dual platform cooperative detection system constructed in this paper has the advantages of a bistatic radar system and single radar system [25,26,27]. The detection range was extended and the detection performance improved, which lays a foundation for future research of the airborne radar cooperative detection system.

The rest of this paper is organized as follows. In Section 2, the system configuration model of cooperative illumination is established, and the echo model of the system is derived by taking linear frequency modulation (LFM) signal as an example. The signal processing and fusion detection flow of the cooperative detection system is designed, and the theoretical derivation and numerical simulation are carried out in Section 3. Section 4 verifies the feasibility and effectiveness of the signal processing and fusion detection algorithm by the simulation experiment of the designed cooperative detection system. Conclusions are presented in Section 5.

The notations used in this paper are shown in Table 1.

## 2. Modeling

**Figure 1:**
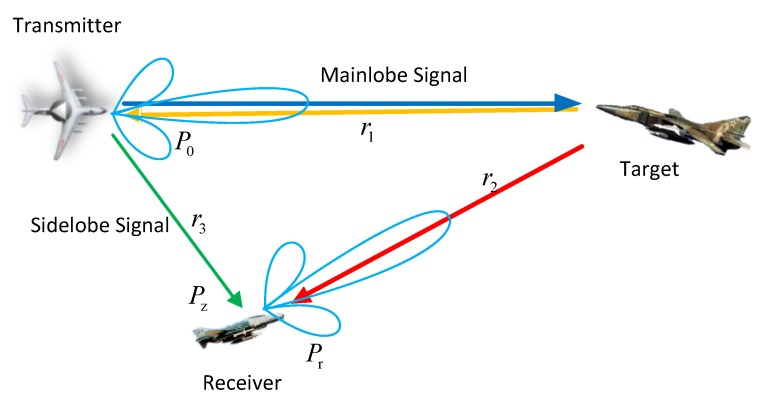
Scene schematic diagram of cooperative irradiation.

The scene relationship between the airborne cooperative detection system and target is shown in Figure 1, in which the AEW and the UAV constitute a dual platform cooperative detection system, and the AEW is used as a transmitter and receiver (T/R), and the UAV is used as a receiver (R). The working process of the cooperative system can be expressed as follows: Firstly, the AEW transmits signal to irradiate target Tg. Then, target Tg reflects the signal. Finally, AEW and UAV will receive the target echo, respectively. The AEW (T/R) is a single radar system, and then the AEW (T) and the UAV form a bistatic radar system. In fact, the side lobe signal of AEW will also be directly received by UAV (R), which means that the echo signal received by the UAV includes both the target reflection signal and the side lobe direct wave signal. In Figure 1, the target echo power received by T/R is denoted as $P_{0}$, the power of the target echo signal reaching the R is denoted as $P_{r}$, the power of the AEW side lobe signal reaching the R directly is denoted as $P_{z}$, $r_{1}$ is the distance from T/R to Tg, $r_{2}$ is the distance from Tg to R, and $r_{3}$ is the distance from T/R to R.

**Figure 2:**
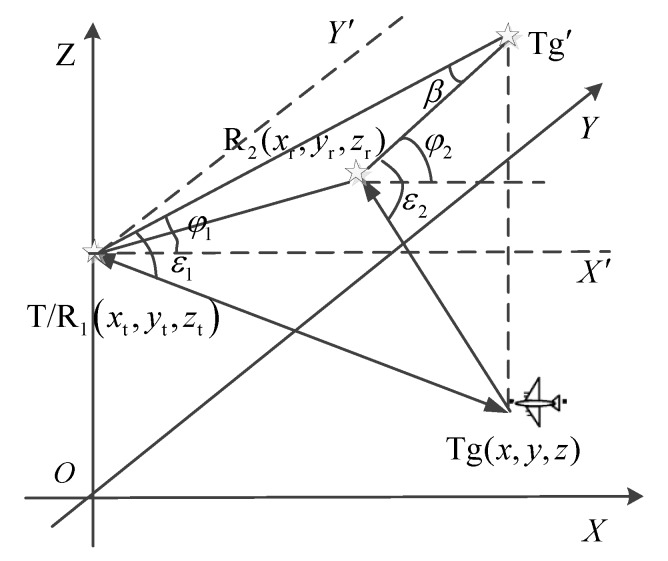
Geometric diagram of the cooperative illumination scene.

Further, the Cartesian coordinate system is established by taking the position of the transmitter as the origin and taking the transmitter-to-receiver connection as the *X*-axis. The *Y*-axis and the *Z*-axis are perpendicular to the *X*-axis, satisfying the right-hand criterion. The geometric relationship between the transmitter, receiver, and target is shown in Figure 2. The coordinates of the transmitter are $r_{t}\left(x_{t},y_{t},z_{t}\right)$, that of the receiver are $r_{r}\left(x_{r},y_{r},z_{r}\right)$, and that of the target are $r_{\mathrm{tg}}\left(x,y,z\right)$. The azimuth angle of the target relative to the transmitter is denoted as $\varphi_{1}$, where the pitch angle is denoted as $\varepsilon_{1}$ and the azimuth angle of the target relative to the receiver is denoted as $\varphi_{2}$, where the pitch angle is denoted as $\varepsilon_{2}$.

According to the cooperative detection system geometric model, the expression of the signal model is derived.

The method in this paper is suitable for LFM waveforms and coded signal. In this paper, the LFM signal is selected for echo signal derivation and simulation. 

Assuming that the radar transmits LFM signal, the signal can be expressed as:

(1) $$s\left(t\right)=\mathrm{rect}\left(\frac{t_{f}}{T_{p}}\right)\exp\left(j2\pi \left(f_{c}t+\frac{1}{2}Kt_{f}^{2}\right)\right),$$

where rect(·) is the rectangle function; $f_{c}$ is the carrier frequency; $T_{p}$ is the pulse width; K is the frequency modulation slope, $K=\frac{B}{T_{p}}$; B is the signal bandwidth; and $t_{f}$ is the fast-time. Then, the corresponding echo signal can be expressed as:

(2) $$s_{r}\left(t_{f},\tau \right)=\mathrm{rect}\left(\frac{t_{f}-\tau }{T_{p}}\right)\cdot \exp\left(j2\pi f_{c}\tau \right)\cdot \exp\left(j\pi K{\left(t_{f}-\tau \right)}^{2}\right),$$

where τ is the delay.

For a single radar system with an integrated transceiver and receiver, the delay is expressed as:

(3) $$\tau_{a}=\frac{2\left(r_{1}+v_{1}t_{n}\right)}{c},$$

where $t_{n}$ is the slow-time and $v_{1}$ is the radial velocity between the transmitter and the target, which is given by:

(4) $$v_{1}=\left({\vec{v}}_{\mathrm{tg}}-{\vec{v}}_{t}\right)\cdot \left(\frac{{\vec{r}}_{\mathrm{tg}}-{\vec{r}}_{t}}{\left|{\vec{r}}_{\mathrm{tg}}-{\vec{r}}_{t}\right|}\right),$$

where ${\vec{v}}_{t}$ is the transmitter velocity vector and ${\vec{v}}_{tg}$ is the target velocity vector.

The target echo signal of a single radar system is:

(5) $$\begin{array}{ll} s_{\mathrm{ra}}\left(t_{f},t_{n}\right) & =\mathrm{rect}\left(\frac{t_{f}-\tau_{a}}{T_{p}}\right)\cdot \exp\left(j2\pi f_{c}\tau_{a}\right)\cdot \exp\left(j\pi K{\left(t_{f}-\tau_{a}\right)}^{2}\right)\\ & =\mathrm{rect}\left[\frac{1}{T_{p}}\left(t_{f}-\frac{2\left(r_{1}+v_{1}t_{n}\right)}{c}\right)\right]\cdot \exp\left[j2\pi f_{c}\left(\frac{2\left(r_{1}+v_{1}t_{n}\right)}{c}\right)\right]\\ & \cdot \exp\left[j\pi K{\left(t_{f}-\frac{2\left(r_{1}+v_{1}t_{n}\right)}{c}\right)}^{2}\right] \end{array}$$

Then, the final echo of a single radar receiver can be expressed as:

(6) $$S_{r0}=s_{\mathrm{ra}}\left(t_{f},t_{n}\right)+\sigma_{n}\cdot s_{n},$$

where $\sigma_{n}$ is the noise amplitude and $\sigma_{n}=\frac{1}{\sqrt{10^{SNR/10}}}$, SNR is the signal-to-noise ratio, and $s_{n}$ is the white noise.

For bistatic radar systems, the time delay is:

(7) $$\tau_{b}=\frac{r_{1}+v_{1}t_{n}+r_{2}+v_{2}t_{n}}{c},$$

where $v_{2}$ is the radial velocity between the target and the receiver, expressed as:

(8) $$\tau_{b}=\frac{r_{1}+v_{1}t_{n}+r_{2}+v_{2}t_{n}}{c},$$

where ${\vec{v}}_{r}$ is the receiver velocity vector.

Then, the target echo signal of a bistatic radar system is:

(9) $$\begin{array}{ll} s_{\mathrm{rb}}\left(t_{f},t_{n}\right) & =\mathrm{rect}\left(\frac{t_{f}-\tau_{b}}{T_{p}}\right)\cdot \exp\left(j2\pi f_{c}\tau_{b}\right)\cdot \exp\left(j\pi K{\left(t_{f}-\tau_{b}\right)}^{2}\right)\\ & =\mathrm{rect}\left[\frac{1}{T_{p}}\left(t_{f}-\frac{r_{1}+v_{1}t_{n}+r_{2}+v_{2}t_{n}}{c}\right)\right]\cdot \exp\left[j2\pi f_{c}\left(\frac{r_{1}+v_{1}t_{n}+r_{2}+v_{2}t_{n}}{c}\right)\right]\\ & \cdot \exp\left[j\pi K{\left(t_{f}-\frac{r_{1}+v_{1}t_{n}+r_{2}+v_{2}t_{n}}{c}\right)}^{2}\right] \end{array}$$

The time delay of the direct wave signal is:

(10) $$\tau_{d}=\frac{r_{3}+v_{3}t_{n}}{c},$$

where $v_{3}$ is the radial velocity between the transmitter and receiver, expressed as:

(11) $$v_{3}=\left({\vec{v}}_{r}-{\vec{v}}_{t}\right)\cdot \left(\frac{{\vec{r}}_{r}-{\vec{r}}_{t}}{\left|{\vec{r}}_{r}-{\vec{r}}_{t}\right|}\right).$$

Then, the direct wave signal received by the receiver can be expressed as:

(12) $$\begin{array}{ll} s_{\mathrm{rd}}\left(t_{f},t_{n}\right) & =\mathrm{rect}\left(\frac{t_{f}-\tau_{d}}{T_{p}}\right)\cdot \exp\left(j2\pi f_{c}\tau_{d}\right)\cdot \exp\left(j\pi K{\left(t_{f}-\tau_{d}\right)}^{2}\right)\\ & =\mathrm{rect}\left[\frac{1}{T_{p}}\left(t_{f}-\frac{r_{3}+v_{3}t_{n}}{c}\right)\right]\cdot \exp\left[j2\pi f_{c}\left(\frac{r_{3}+v_{3}t_{n}}{c}\right)\right]\\ & \cdot \exp\left[j\pi K{\left(t_{f}-\frac{r_{3}+v_{3}t_{n}}{c}\right)}^{2}\right] \end{array}$$

The final echo of the receiver can be expressed as:

(13) $$S_{r1}=s_{\mathrm{rb}}\left(t_{f},t_{n}\right)+\sigma_{z}\cdot s_{\mathrm{rd}}\left(t_{f},t_{n}\right)+\sigma_{n}\cdot s_{n},$$

where $\sigma_{z}$ is the amplitude of the direct wave and $\sigma_{z}=\frac{1}{\sqrt{10^{SJR/10}}}$, and SJR is the signal-to-jamming ratio.

## 3. Target Cooperative Detection for the Cooperative Detection System.

**Figure 3:**
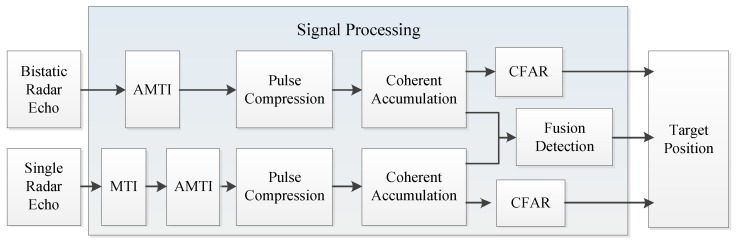
Block diagram of the radar echo processing for the cooperative detection system.

According to the cooperative detection system model mentioned above, in this section, the echo simulation of single radar system and bistatic radar system was carried out. Then, the signal was processed. The constant false alarm (CFAR) processor was carried out to obtain the detection results. Finally, the target was located according to the detection results. Meanwhile, in order to improve the detection performance, the echo results of the two receivers were fused to obtain the detection results. The block diagram of the processing process is shown in Figure 3.

Without loss of generality, the following assumptions were made before introducing the cooperative detection method:

(1) The receiving platform and the transmitting platform have their own navigation system, which can get their own position information in real time and communicate with each other.
(2) The target is not at the base line of the transceiver and receiver platform, which means there is a time delay between the direct wave and the echo arriving at the receiver.
(3) The main lobe direction of the radar antenna is known, which is a three-dimensional radar.
(4) The arrival time of the echo can be measured.

### 3.1. Detection Range Comparison

According to the preset scene, the power expression of the target echo signal arriving at the radar receiver of the AEW is deduced as:

(14) $$P_{0}=\frac{P_{t}G_{t}^{2}\sigma \lambda^{2}}{{\left(4\pi \right)}^{3}r_{1}^{4}L_{1}L_{e}}=P_{t}+2G_{t}+\sigma +2\lambda -33-4r_{1}-L_{1}-L_{e},$$

where $P_{t}$ is the radar transmitting peak power, $G_{t}$ is the gain of the radar transmitting antenna in the target direction, σ is the radar sectional area of the target in the direction of the AEW, λ is the working wavelength of the radar, $L_{1}$ is the radar receiver loss of the AEW, and $L_{e}$ is other losses (transmission loss, atmospheric loss, pulse pressure loss, etc.).

The AEW radar transmits signals. Then, the signals reach the receiver radar after the target reflection. The power received by the receiver radar is expressed as:

(15) $$P_{r}=\frac{P_{t}G_{t}G_{r}\sigma^{\prime }\lambda^{2}}{{\left(4\pi \right)}^{3}r_{1}^{2}r_{2}^{2}L_{2}{L^{\prime }}_{e}}=P_{t}+G_{t}+G_{r}+\sigma^{\prime }+2\lambda -33-2r_{1}-2r_{2}-L_{2}-{L^{\prime }}_{e},$$

where $G_{r}$ is the main lobe gain of the passive receiver antenna, $\sigma^{\prime }$ is the radar cross-sectional area of the target in the direction of the receiver (bistatic radar cross-sectional area), $L_{2}$ is the passive receiver loss, and ${L^{\prime }}_{e}$ is other losses.

The power expression of the radar side lobe signal received by the receiver radar from the AEW is as follows:

(16) $$P_{z}=\frac{P_{t}{G^{\prime }}_{t}A_{r}}{4\pi r_{3}^{2}L_{2}{L^{\prime \prime }}_{e}}=\frac{P_{t}{G^{\prime }}_{t}{G^{\prime }}_{r}\lambda^{2}}{{\left(4\pi r_{3}\right)}^{2}L_{2}{L^{\prime \prime }}_{e}}=P_{t}+{G^{\prime }}_{t}+{G^{\prime }}_{r}+2\lambda -22-2r_{3}-L_{2}-{L^{\prime \prime }}_{e},$$

where $A_{r}$ is the effective receiving area of the receiver, ${G^{\prime }}_{t}$ is the sidelobe gain of the AEW radar transmitting antenna, ${G^{\prime }}_{r}$ is the sidelobe gain of the receiver antenna, and ${L^{\prime \prime }}_{e}$ is other losses.

According to the above expression, the ratio of the target echo power of the receiver to the target echo power of the radar receiver of the AEW can be expressed as:

(17) $$K_{1}=\frac{P_{r}}{P_{0}}=\left(\frac{P_{t}G_{t}G_{r}\sigma^{\prime }\lambda^{2}}{{\left(4\pi \right)}^{3}r_{1}^{2}r_{2}^{2}L_{2}{L^{\prime }}_{e}}\right)/\left(\frac{P_{t}G_{t}^{2}\sigma \lambda^{2}}{{\left(4\pi \right)}^{3}r_{1}^{4}L_{1}L_{e}}\right)=\frac{G_{r}r_{1}^{2}\sigma^{\prime }L_{1}L_{e}}{G_{t}r_{2}^{2}\sigma L_{2}{L^{\prime }}_{e}}.$$

In order to analyze the main factors affecting the ratio, $K_{1}$, assuming $\frac{L_{1}L_{e}}{L_{2}{L^{\prime }}_{e}}≅1$, there is:

(18) $$K_{1}=\frac{P_{r}}{P_{0}}\approx \frac{G_{r}r_{1}^{2}\sigma^{\prime }}{G_{t}r_{2}^{2}\sigma }.$$

If the main lobe gain of the radar antenna of the receiver, $G_{r}$, is equal to that of the radar antenna of the AEW, $G_{t}$, the cross-section area of the bistatic radar is larger than that of the single radar, and the distance between the receiver and the target is smaller than that between the AEW and the target, i.e., $K_{1}\geq 1$. According to the formula, when $K_{1}\geq 1\left(K_{1}\geq 0\mathrm{dB}\right)$, the receiving echo power of the receiver is greater than or equal to the receiving echo power of the AEW. If the processing gain of the approximate matched filtering can be obtained in the process of signal detection, the detection ability of the bistatic system is equal to that of the active detection ability of the AEW radar, and the target can be detected at this time.

**Figure 4:**
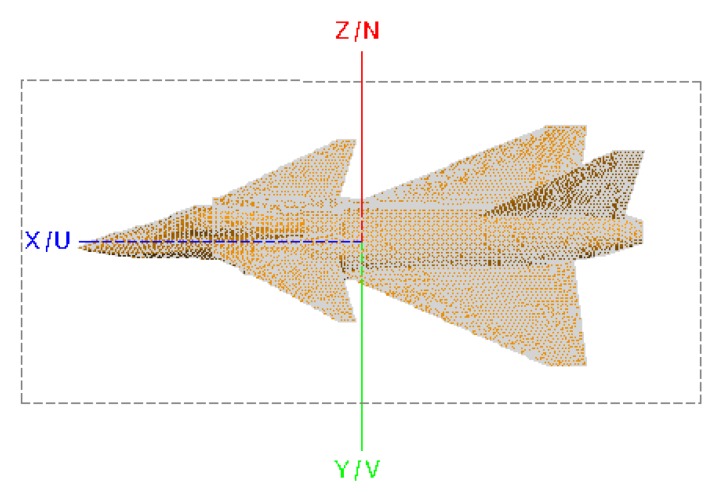
Target model and mesh generation.

**Figure 5:**
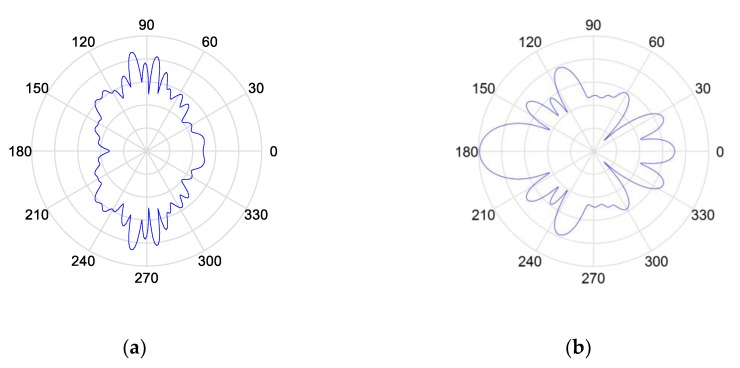
Simulation of the radar cross-section: (**a**) Cross-section of the single radar; (**b**) Cross-section of the bistatic radar.

In this paper, FEKO was used to simulate the cross-sectional area of the single radar and bistatic radar. The target model is shown in Figure 4, and the simulation results of the cross-sectional area of the single radar and bistatic radar are shown in Figure 5.

**Figure 6:**
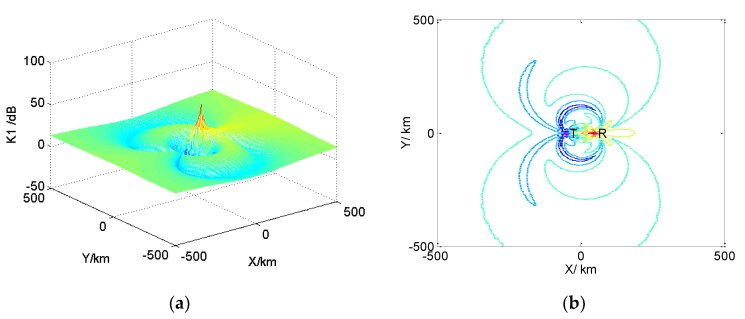
The value of the distribution of $K_{1}$: (**a**) Three-dimensional distribution of $K_{1}$; (**b**) The isogram of the distribution of $K_{1}$.

According to the above formula derivation and radar sectional area, numerical simulation analysis was conducted, and the results are shown in Figure 6. 

Figure 6 is the value distribution diagram of the ratio, $K_{1}$, Figure 6a is the three-dimensional diagram of the value distribution of $K_{1}$, and Figure 6b is the isogram of the value distribution of $K_{1}$. It can be seen from the figures that in the range of 500 km, except for some positions near the launch platform, the receiving power of the bistatic radar is less than that of the AEW radar, and other positions are larger than that of the AEW radar. That is to say, the distance of bistatic detectable targets is farther than that of AEW, namely the detection performance of the bistatic radar is better than that of AEW. At the same time, it can be seen from the figure that the location distribution of $K_{1}<0\;\;\mathrm{dB}$ is close to the launching platform. For the cooperative detection of AEW and UAV, the AEW is located in the rear of the security, the receiver goes forward and receives the signal silently, and the detection performance of the target in the detection area is slightly affected. The detection range of the two-platform cooperative detection system is extended, and the detection range is complementary to each other, so it has more advantages in target detection.

### 3.2. Direct Wave Suppression

The ratio of receiver target echo to direct wave power can be expressed as:

(19) $$K_{2}=\frac{P_{r}}{P_{z}}=\left(\frac{P_{t}G_{t}G_{r}\sigma^{\prime }\lambda^{2}}{{\left(4\pi \right)}^{3}r_{1}^{2}r_{2}^{2}L_{2}{L^{\prime }}_{e}}\right)/\left(\frac{P_{t}{G^{\prime }}_{t}{G^{\prime }}_{r}\lambda^{2}}{{\left(4\pi r_{3}\right)}^{2}L_{2}{L^{\prime \prime }}_{e}}\right)=\frac{G_{t}G_{r}r_{3}^{2}\sigma^{\prime }{L^{\prime \prime }}_{e}}{4\pi {G^{\prime }}_{t}{G^{\prime }}_{r}r_{1}^{2}r_{2}^{2}{L^{\prime }}_{e}}.$$

The power ratio of target echo to direct wave can be estimated under different conditions.

In order to analyze the main factors affecting the ratio, $K_{2}$, assuming $\frac{L_{e}}{{L^{\prime }}_{e}}≅1$, there is:

(20) $$K_{2}=\frac{P_{r}}{P_{z}}\approx \frac{1}{4\pi }\frac{G_{r}G_{t}r_{3}^{2}\sigma^{\prime }}{G_{r}{}^{\prime }G_{t}{}^{\prime }r_{1}^{2}r_{2}^{2}}=M\frac{r_{3}^{2}\sigma^{\prime }}{4\pi r_{1}^{2}r_{2}^{2}},$$

where $M=\frac{G_{r}G_{t}}{G_{r}{}^{\prime }G_{t}{}^{\prime }}$.

**Figure 7:**
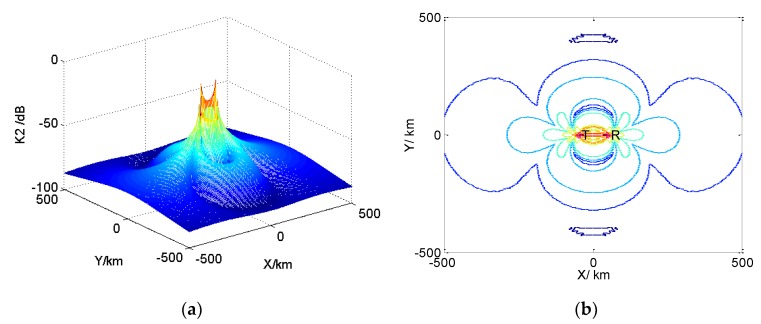
The value of distribution of $K_{2}$: (**a**) Three-dimensional distribution of $K_{2}$; (**b**) The isogram of the distribution of $K_{2}$.

It was assumed that the other losses of the AEW and the receiver are similar, and the processing gain of the approximate matched filter can be obtained in the process of signal detection for the received target echo signal. Any M=44  dB was selected, and $K_{2}$ was simulated at different target positions according to the cross-sectional area of the bistatic radar. The results are shown in Figure 7, where Figure 7a is the three-dimensional diagram of the value distribution of the ratio, and Figure 7b is the isogram of the value distribution of the ratio. As can be seen from the figure, in the simulation area, the ratio is the highest at the location of the transceiver platform, which is −11 dB, while the other locations are basically below −30 dB, namely $K_{2}\ll 1$. The power of the direct wave is far greater than that of the bistatic radar receiver. If the direct wave is not processed, the direct wave signal will enter the target echo channel from the antenna side lobe of the receiver to form false target interference, and at the same time, the detection threshold will be raised, and the target signal cannot be detected.

### 3.3. CFAR Detection

**Figure 8:**
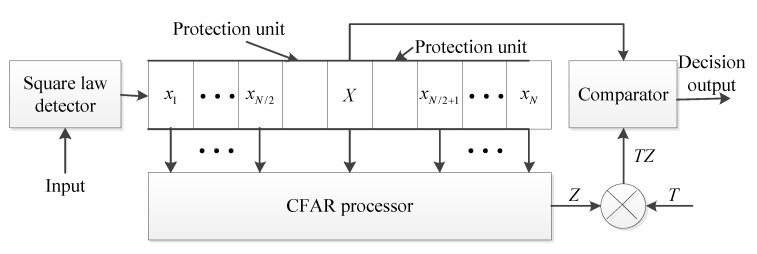
CFAR processor architecture.

The number of reference units detected by CFAR is N. The distribution of reference units detected by CFAR is shown in Figure 8. In the figure, X is the unit to be tested. The left and right units of X are set as protection units, with N/2 reference units on the left and right.

According to N reference units, the CFAR processor can get a relative estimation of the background intensity, Z, which is related to the CFAR detection method. In the multiplier, the decision threshold, TZ, can be obtained by multiplying the estimated value, Z, by a threshold weighting coefficient, T, where $T=Np_{f}{}^{-1/N-1}$ and $p_{f}$ is the false alarm probability. In the comparator, the detected unit is compared with the decision threshold, TZ. If X is larger than TZ, the output is 1; otherwise, the output is 0. 

In the CFAR detector, there are two methods to calculate the signal evaluation, Z, of N reference units: The CA-CFAR detection method and the OS-CFAR detection method. CA-CFAR has good detection performance in a uniform environment, and OS-CFAR has obvious advantages in a clutter edge and multi-target environment. 

In the CA-CFAR detector, Z is the average value of all reference unit signals, $x_{i},i=1,\cdots ,N$:

(21) $$Z=\frac{1}{N}\sum_{i=1}^{N}x_{i}.$$

In the OS-CFAR detector, by sorting the signals, $x_{i},i=1,\cdots ,N$, of N reference units according to amplitude from large to small, one of the order values is taken as the background noise to estimate Z, then:

(22) $$\begin{array}{l} x_{\left(1\right)}\geq x_{\left(2\right)}\geq \cdots \geq x_{\left(k\right)}\geq \cdots \geq x_{\left(N\right)}\\ Z=x_{\left(k\right)} \end{array}$$

### 3.4. Fusion Detection Probability

**Figure 9:**
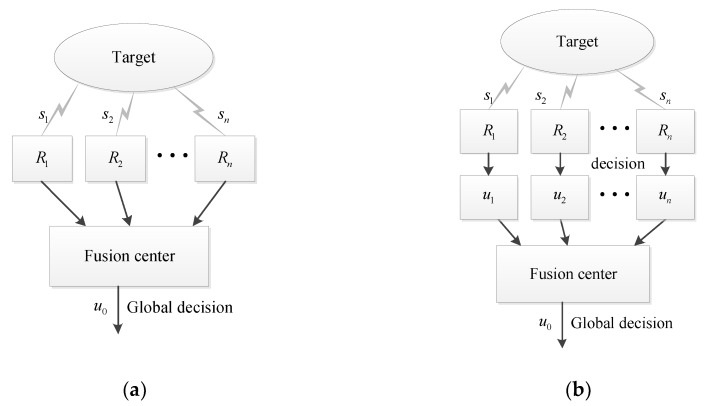
Topology structure diagram of fusion detection: (**a**) Measurement fusion; (**b**) Decision fusion.

The cooperative detection system mentioned in this paper can form a single radar system and bistatic radar system. The fusion detection can be carried out in different processing units, which can be divided into measurement fusion and decision fusion. Figure 9 shows the topology structure of fusion detection. In Figure 9a, measurement fusion is that each receiver can observe independently to get the observation matrix, and then a fusion center receives the observation information of receivers for judgment. In Figure 9b, decision fusion is that each receiver observes and judges independently, and then a fusion center receives the decision information of receivers, and a global decision is obtained.

For measurement fusion, after CFAR detection in the signal processing flow, the measurement matrices of the systems can be obtained respectively, namely, $R_{1},R_{2},\cdots ,R_{n}$. The matrices can be superposed and fused. Then, the existence of the target can be determined, and the detection probability can be calculated through multiple tests of the Monte-Carlo method.

For decision fusion, the two systems separately detect and decide whether there is a target or not. The detection probability is calculated by the Monte-Carlo method, and the detection probability after the decision is fused. 

The cooperative detection system is composed of n receivers $R_{i}\left(i=1,2,\cdots ,n\right)$, and the detection probability of a target is $P\left(R_{i}\right)$. 

The OR-like fusion rule can be expressed as:

(23) $$\begin{array}{cc} u_{0}=\sum_{i=1}^{n}u_{i}, & \{\begin{array}{cc} u_{0}=1 & u_{0}\geq 1\\ u_{0}=0 & u_{0}<1 \end{array} \end{array}.$$

The total probability of simultaneous detection of the target by each receiver is:

(24) $$P_{sor}=P\left(R_{1}\cup R_{2}\cup \cdots R_{n}\right)=1-P\left({\bar{R}}_{1}\right)P\left({\bar{R}}_{2}\right)\cdots P\left({\bar{R}}_{n}\right).$$

Therefore, the AND-like fusion rule can be expressed as:

(25) $$\begin{array}{cc} u_{0}=\prod_{i=1}^{n}u_{i}, & \{\begin{array}{cc} u_{0}=1 & u_{0}\geq 1\\ u_{0}=0 & u_{0}<1 \end{array} \end{array}.$$

The total probability of simultaneous detection of the target by each receiver is:

(26) $$P_{sand}=P\left(R_{1}\cap R_{2}\cap \cdots R_{n}\right)=P\left(R_{1}\right)P\left(R_{2}\right)\cdots P\left(R_{n}\right).$$

The cooperative detection system in this paper is composed of a single base system and double base system. The detection probability of two receivers is $P\left(R_{1}\right)$ and $P\left(R_{2}\right)$, respectively. Then, for OR-like fusion rule, the fusion detection probability can be expressed as

(27) $$P_{sor}=1-P\left({\bar{R}}_{1}\right)P\left({\bar{R}}_{2}\right)$$

For the AND-like fusion rule, the fusion detection probability can be expressed as:

(28) $$P_{s\mathrm{and}}=P\left(R_{1}\right)P\left(R_{2}\right)$$

Now, we analyzed the complexity of the two algorithms. Without loss of generality, one-time multiplication or one-time addition is regarded as a basic calculation amount, and the sum of all addition and multiplication times in an algorithm is regarded as the basic operation amount of the algorithm [28]. The process of the measurement-fusion algorithm and decision-fusion algorithm is the same before getting the measurement vector, so the complexity analysis of this paper starts from the measurement vector. Assuming that the length of measurement vector is M, the number of fusion sensors is two, the number of CFAR detection reference units is N, and the number of Monte-Carlo experiments is $n_{l}$. For measurement fusion, firstly, the measurement vector is fused. The complexity of the OR-like rule is the same as the AND-like rule, which is recorded as O(M). Then, the fusion vector is judged. First, the reference threshold is calculated. The mean value of reference cells is calculated by the CA-CFAR algorithm, with the complexity of O(MN). The reference cells are sorted by the OS-CFAR algorithm, with the complexity of $O\left(MN^{2}\right)$. Then, whether there is a target is judged, with the complexity of O(M). For decision fusion, the measurement vectors are judged respectively. First, the reference threshold is calculated, and the average value of the reference units is calculated by the CA-CFAR algorithm, with the complexity of O(2MN). The reference units are ordered by the OS-CFAR algorithm, with the complexity of $O\left(2MN^{2}\right)$. Then, whether there is a target is judged, and the complexity of the decision is O(2M). Then, the decision vectors are fused, or the complexity of the criteria is the same as that of the criteria, which is recorded as O(M). Finally, the Monte-Carlo experiment is carried out, and the complexity coefficient is $n_{l}$. The total computational complexity of different fusion methods combined with different CFAR algorithms is shown in Table 2. It can be seen that the computation of the measurement fusion is slightly less than that of the decision fusion, and the computation of OS-CFAR is slightly more than that of CA-CFAR.

### 3.5. Estimation of Target Position Parameters

In the scenario assumed in this paper, the position of ${T/R}_{1}\left(x_{t},y_{t},z_{t}\right)$ and $R_{2}\left(x_{r},y_{r},z_{r}\right)$ is known, and the baseline length, $r_{3}$, is known, expressed as:

(29) $$r_{3}=\sqrt{{\left(x_{t}-x_{r}\right)}^{2}+{\left(y_{t}-y_{r}\right)}^{2}+{\left(z_{t}-z_{r}\right)}^{2}}.$$

The delay time of the direct wave echo, $t_{1}$, can be estimated:

(30) $$t_{1}=\frac{r_{3}}{c},$$

where $c=3\times 10^{8}m/s$ is the propagation velocity of the electromagnetic wave.

Converted to the corresponding distance unit, then:

(31) $$R_{g1}=\frac{ct_{1}}{2},$$

(32) $$N_{z}=\frac{2\left(R_{g1}-R_{\min}\right)}{cT_{s}},$$

where $T_{s}$ is the sampling time period, $R_{\min}$ is the minimum detection distance and $R_{g1}$ is the false target range parameter.

According to the geometric relationship in Figure 2, the following equation can be obtained:

(33) $$\{\begin{array}{c} r_{1}=\sqrt{{\left(x_{t}-x\right)}^{2}+{\left(y_{t}-y\right)}^{2}+{\left(z_{t}-z\right)}^{2}}\\ \begin{array}{l} r_{2}=\sqrt{{\left(x_{r}-x\right)}^{2}+{\left(y_{r}-y\right)}^{2}+{\left(z_{r}-z\right)}^{2}}\\ \tan\varphi_{1}=\frac{y-y_{t}}{x-x_{t}}\\ \sin\varepsilon_{1}=\frac{z-z_{t}}{r_{1}} \end{array} \end{array},$$

where $\varphi_{1}$ and $\varepsilon_{1}$ are the azimuth angle and pitch angle corresponding to the center line of tthte transmitter antenna main lobe, respectively.

The direct wave time is expressed as $t_{1}$ and the echo time is expressed as $t_{2}$, then:

(34) $$c\left(t_{2}-t_{1}\right)=r_{1}+r_{2}-r_{3}.$$

By solving Equations (33) and (34), the location information of the target can be obtained, and the target location can be carried out according to the estimated parameters.

## 4. Simulation Analysis of Target Cooperative Detection

According to the commonly used data range of the existing equipment working mode, simulation parameters were set. The parameters are shown in Table 3.

### 4.1. Analysis of Simulation Results of Single-Base Echo

**Figure 10:**
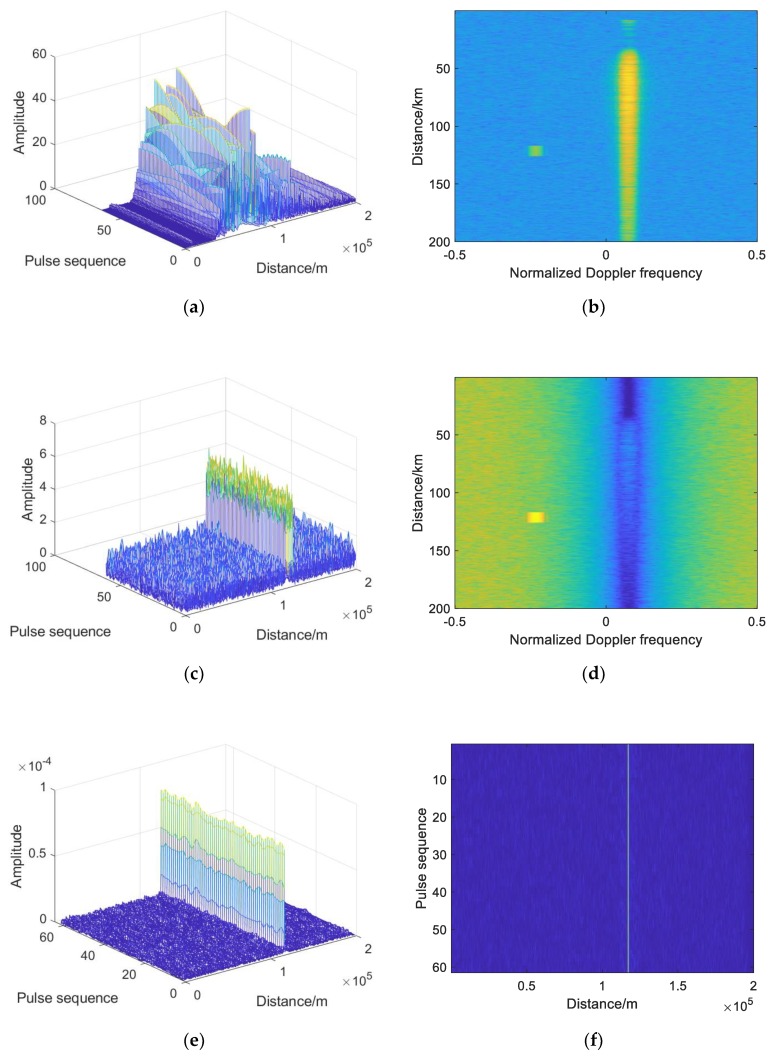

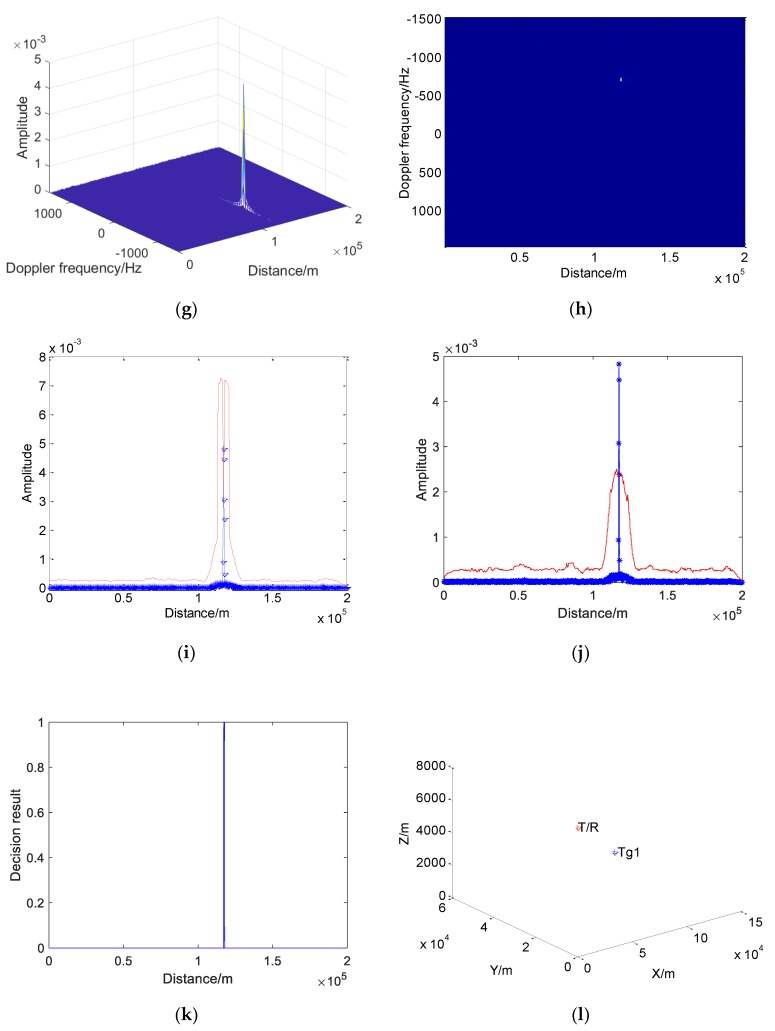
Simulation results of single base radar target echo: (**a**) Three-dimensional information of the echo signal; (**b**) Spectrum of the echo signal; (**c**) Echo signal processed by AMTI; (**d**) Spectrum of the echo signal processed by AMTI; (**e**) Three-dimensional of the echo signal processed by pulse compression; (**f**) Two-dimensional of the echo signal processed by pulse compression; (**g**) Three-dimensional of the echo signal after phase-coherent accumulation processing; (**h**) Two-dimensional of the echo signal after phase-coherent accumulation processing; (**i**) Result of CA-CFAR test; (**j**) Result of OS-CFAR test; (**k**) Decision result; (**l**) The position of target coordinate.

According to the settings and simulation parameters, the simulation results of echo and signal processing of single-base radar system are shown in Figure 10. Figure 10a is the three-dimensional information of the received echo, in which the target signal is completely submerged. Figure 10b is the spectrum of the echo signal. It can be seen that the echo Doppler frequencies are mainly concentrated in two places, one of which exists from 50 to 200 km, and the power is high, which is the clutter Doppler frequency generated by the platform motion. The other is 120 km, with a small range of distance and Doppler frequency generated by the relative motion of the platform and the target. Figure 10c is the echo signal processed by AMTI. It can be seen that the clutter is well suppressed and the target echo signal appears. Figure 10d is the spectrum of the echo signal processed by AMTI, and the clutter Doppler is basically suppressed. Theoretically, the SNR gain after pulse compression is 14.77 dB, and the two-dimensional and three-dimensional images of the echo signal processed by pulse compression are shown in Figure 10e,f. After pulse compression, the target signal is prominent, and the output SNR is increased. In order to further improve the SNR, the theoretical SNR gain after phase-coherent processing is 18.06 dB. Figure 10g,h are the two-dimensional and three-dimensional images of the echo signal after phase-coherent accumulation processing, and it can be seen that the target signal is more prominent. Figure 10i is the result of the CA-CFAR test, and Figure 10j is the result of the OS-CFAR test. The target signal is detected when it is above the threshold, and Figure 10k is the decision result. The position parameters of the target can be obtained through the geometric relation and radar beam angle. Figure 10l indicates the position of the target coordinate obtained by simulation.

The final positioning error can be obtained by comparing the target position parameters obtained by system detection with the actual positioning parameters. The simulation assumes that the coordinates of the target position are Tgd = [110 40 2] km, and estimates that the target position are Tg1d = [110.1207 40.0439 1.9934] km according to the simulation results; then, the position errors are expressed as Δd = [120.7 43.9 −6.6] m, i.e., 0.11%.

### 4.2. Analysis of Simulation Results of Double-Base Echo

**Figure 11:**
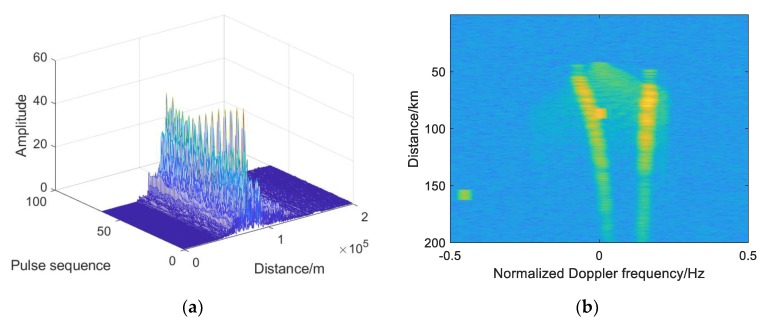

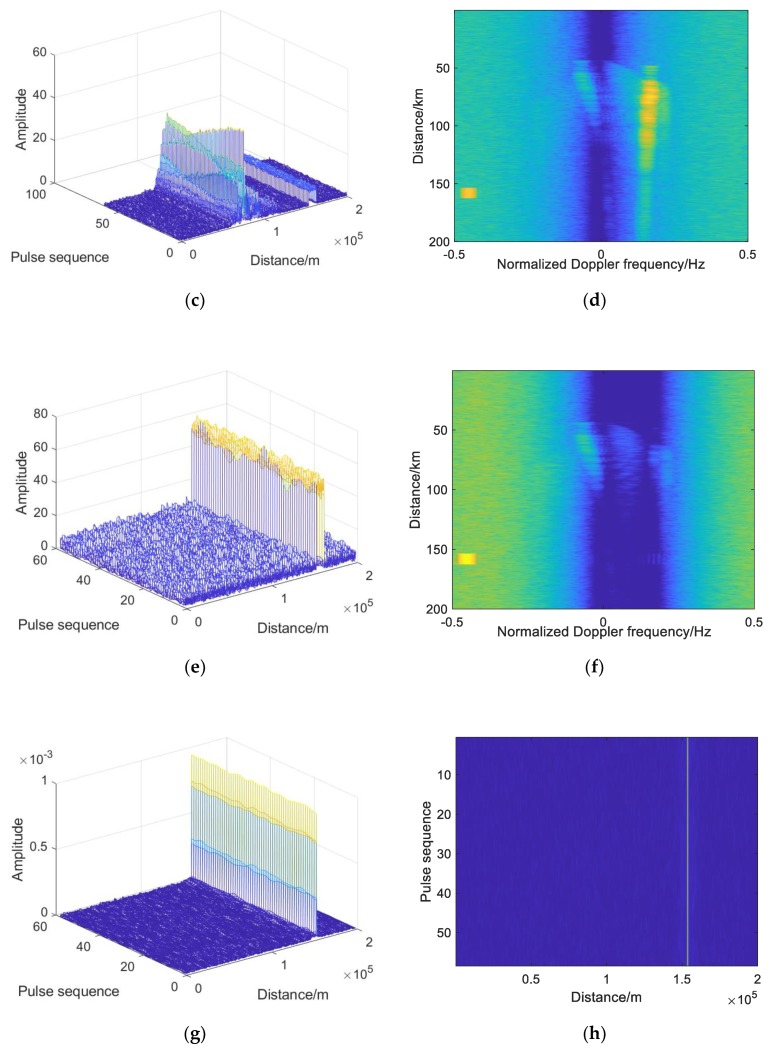

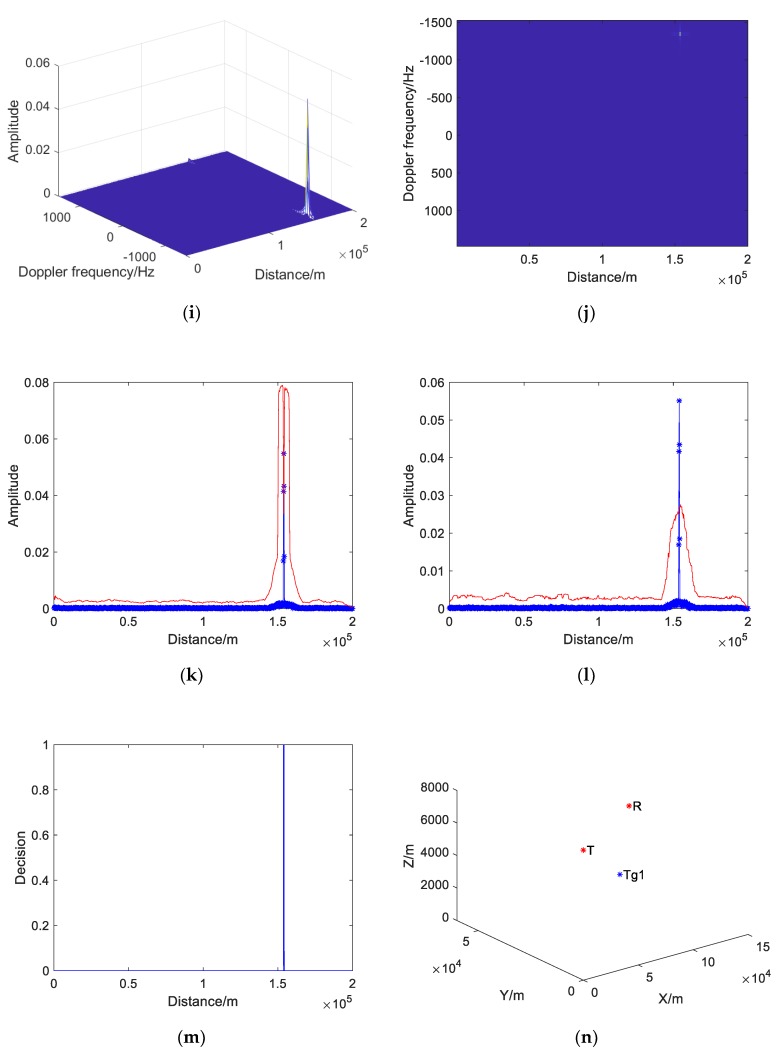
Simulation results of target echo of bistatic radar: (**a**) Three-dimensional information of the echo signal; (**b**) Spectrum of the echo signal; (**c**) Echo signal processed by MTI; (**d**) Spectrum of the echo signal processed by MTI; (**e**) Echo signal processed by AMTI; (**f**) Spectrum of the echo signal processed by AMTI; (**g**) Three-dimensional echo signal processed by pulse compression; (**h**) Two-dimensional echo signal processed by pulse compression; (**i**) Three-dimensional echo signal after phase-coherent accumulation processing; (**j**) Two-dimensional of the echo signal after phase-coherent accumulation processing; (**k**) Result of CA-CFAR test; (**l**) Result of OS-CFAR test; (**m**) Decision result; (**n**) The position of the target coordinate.

According to the set scene and simulation parameters, the simulation results of the echo and signal processing of the bistatic radar system are shown in Figure 11. Figure 11a is the three-dimensional information of the received echo, in which the target signal is completely submerged. Figure 11b is the spectrum of the echo signal. It can be seen that the echo Doppler frequencies are mainly concentrated in four places, two of which exist from 50 to 200 km, and the power is high, which is the clutter Doppler frequency generated by the platform motion. The other two exist in a small range, with the Doppler frequency generated by the relative motion of the platform and the target and the Doppler frequency generated by the direct wave, and the one with higher power is generated by the direct wave. Figure 11c shows the echo signal processed by AMTI. It can be seen that the clutter is well suppressed while the target echo signal appears. Figure 11d is the spectrum of the echo signal processed by AMTI, and the clutter Doppler spectrum is basically suppressed. Figure 11e is an echo signal processed by MTI. It can be seen that the clutter near the target signal is well suppressed and the target echo signal appears. Figure 11f is the spectrum of the echo signal processed by MTI, and the clutter spectrum near zero Doppler is basically suppressed and the direct Doppler spectrum is suppressed. Figure 11g,h are respectively two-dimensional and three-dimensional echo signals processed by pulse compression. After pulse compression, the target signal is prominent, the output SNR is increased, and the direct wave signal is further suppressed. Figure 11i,j are two-dimensional and three-dimensional images of echo signal processed by phase-coherent accumulation. It can be seen that the target signal is more prominent, and the influence of the clutter and direct wave is reduced. Figure 11k is the result of CA-CFAR detection, and Figure 11l is the result of OS-CFAR detection. The target signal is detected when it is above the threshold, and Figure 11m is the decision result. Figure 11n shows the target coordinate position obtained by simulation.

The final positioning error can be obtained by comparing the target position parameters obtained by system detection with the actual positioning parameters. The simulation assumes that the coordinates of the target position are Tgs = [110 40 2] km, and estimates that the target position is Tg1s = [110.099 40.032 1.995] km according to the simulation results; then, the position errors are expressed as Δs = [99 32 −4.9] m, i.e., 0.09%.

### 4.3. Simulation Analysis of CFAR Detection

**Figure 12:**
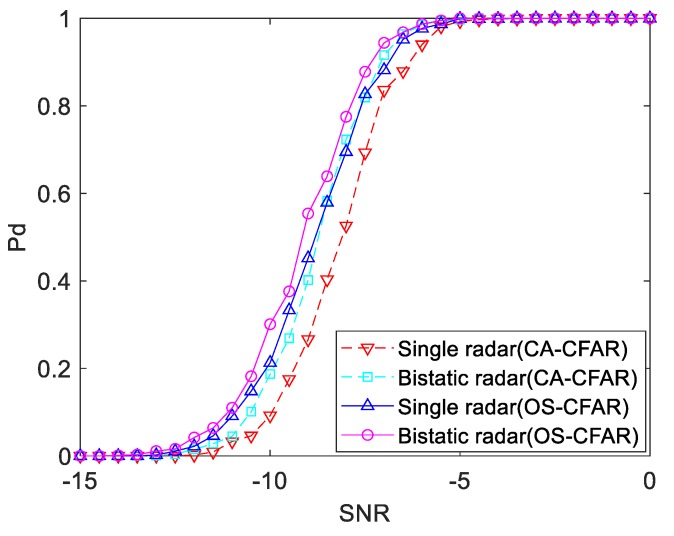
Detection performance curve of the CFAR algorithm.

For the description of CA-CFAR algorithm and OS-CFAR algorithm in Section 3.3, the simulation was performed according to the parameters set previously. The number of reference units in CFAR algorithm was set to 48, and the ordinal value of OS-CFAR was set to 18. Assuming that the echo power of the single radar and bistatic radar at the target position is equal, the Monte-Carlo method was used to calculate the target detection probability under the two algorithms. The simulation results are shown in Figure 12. It can be seen from the figure that when the SNR is −8 dB, the detection probability of the single radar using the CA-CFAR algorithm is 52.6%, and that of the bistatic radar is 72.3%. When using the OS-CFAR algorithm, the detection probability of the single radar is 69.5%, and that of the bistatic radar is 77.5%. That is, under the same SNR, the detection probability of the OS-CFAR algorithm is higher, and it has more advantages in a clutter background. In the design of the distributed architecture in this paper, thte OS-CFAR algorithm is preferred. 

### 4.4. Simulation Analysis of Fusion Detection

According to the simulation results in the previous three sections, it can be found that the detection probability of the bistatic radar system is higher when the SNR is lower than −5 dB. In the background of clutter, the OS-CFAR algorithm has more advantages. In this paper, the local detection adopts the OS-CFAR algorithm. In order to improve the detection probability of the system, the received echo of the single radar and the received echo of the bistatic radar after signal processing were fused for detection. 

From the simulation results of the ratio of the echo power of single-base radar to the target echo power of bistatic radar in Figure 5, it can be known that the value of power is different in different scenarios. According to the simulation parameters set in Section 3.1, the single radar detection results and bistatic radar detection results were fused and simulated by the Monte-Carlo method to analyze the detection probability of the fusion treatment in the following different scenarios.

#### 4.4.1. $K_{1}>1$

**Figure 13:**
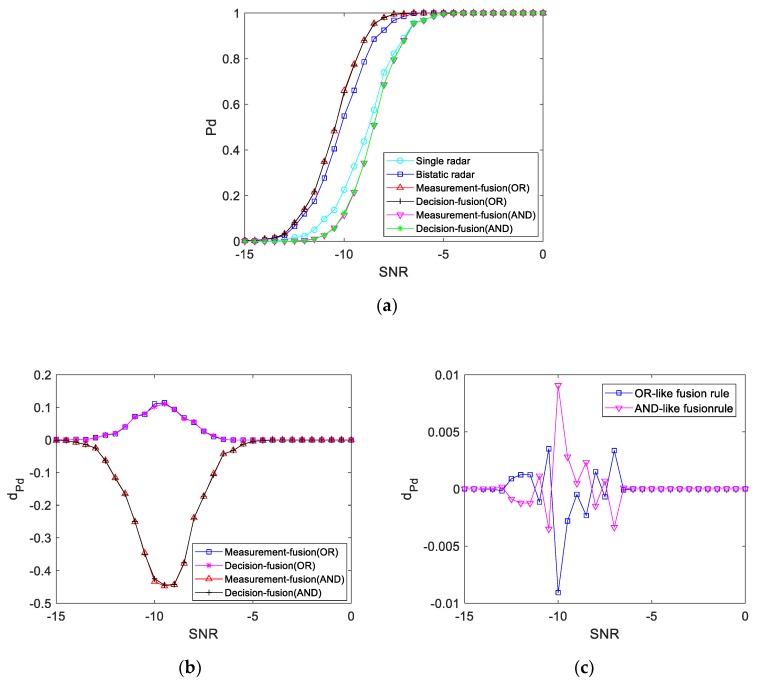
Bistatic radar echo power is greater than single radar echo power: (**a**) Detection performance curve; (**b**) Difference value of the detection performance curve with the bistatic radar; (**c**) Difference value of the fusion detection performance curve.

When the power of the bistatic radar echo at the target position is greater than the power of the single radar echo, the detection performance curve obtained by simulation is shown in Figure 13. It can be seen from the figure that the detection probability of the three systems is different when the SNR is between −15 and −5 dB. When the SNR is −8 dB, the detection probability of the single radar echo is 74%, the detection probability of the bistatic radar echo is 92.5%, the fusion detection probability of the OR-like fusion rule is 97.9%, and the fusion detection probability of the AND-like fusion rule is 68.6%. That is, the detection performance of the double base radar echo is better than that of the single base radar echo at the same SNR, and the detection probability is higher. The detection performance curve of the cooperative detection system after fusion detection of the OR-like fusion rule is much better than that of the single radar system, but it is only slightly better than that of the bistatic radar system. Moreover, the detection performance curve of the cooperative detection system after fusion detection of the AND-like fusion rule is much worse than that of the bistatic radar system, but it is only slightly worse than that of the single radar system. That is to say, when the power of the bistatic radar echo is high, the contribution of the single radar echo signal to fusion detection is limited.

#### 4.4.2. $K_{1}=1$

**Figure 14:**
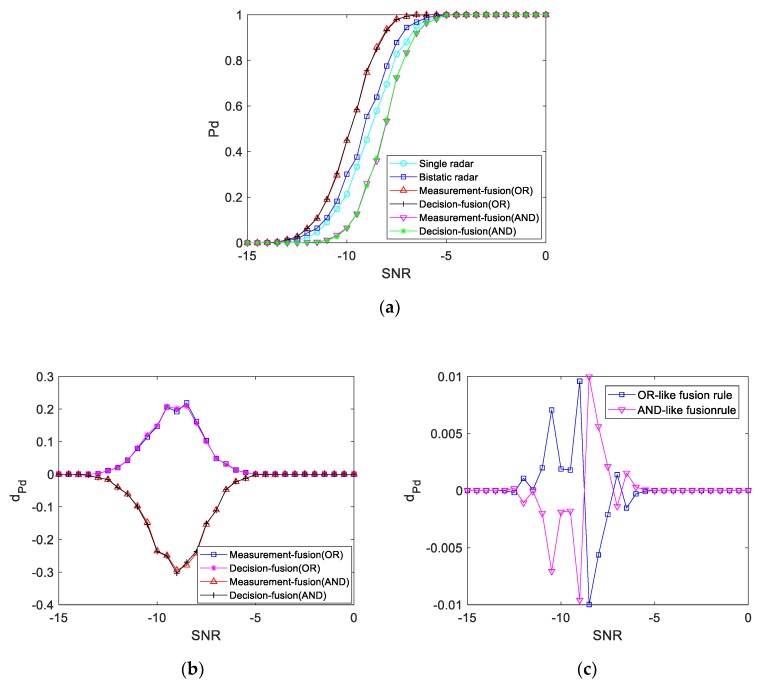
Single and double base target echo signal power is the same: (**a**) Detection performance curve; (**b**) Difference value of the detection performance curve with a bistatic radar; (**c**) Difference value of the fusion detection performance curve.

When the dual-base echo power is the same as the single-base echo power at the target location, the simulation results of the detection performance curve are shown in Figure 14. It can be seen from the figure that the detection probability of the three systems is different when the SNR is between −13 and −5 dB. When the SNR is −8 dB, the detection probability of the single radar echo is 69.5%, the detection probability of the double base echo is 77.5%, the fusion detection probability of the OR-like fusion rule is 93.7%, and the fusion detection probability of the AND-like fusion rule is 53.3%. That is, under the same SNR, the detection performance of the bistatic radar echo is better than that of the single radar echo, and the detection probability is higher. After fusion detection of the OR-like fusion rule, the detection performance curve is better than that of the bistatic radar system and single radar system. Additionally, for the AND-like fusion rule, the detection performance curve is worse than that of the bistatic radar system and single radar system.

#### 4.4.3. $K_{1}<1$

**Figure 15:**
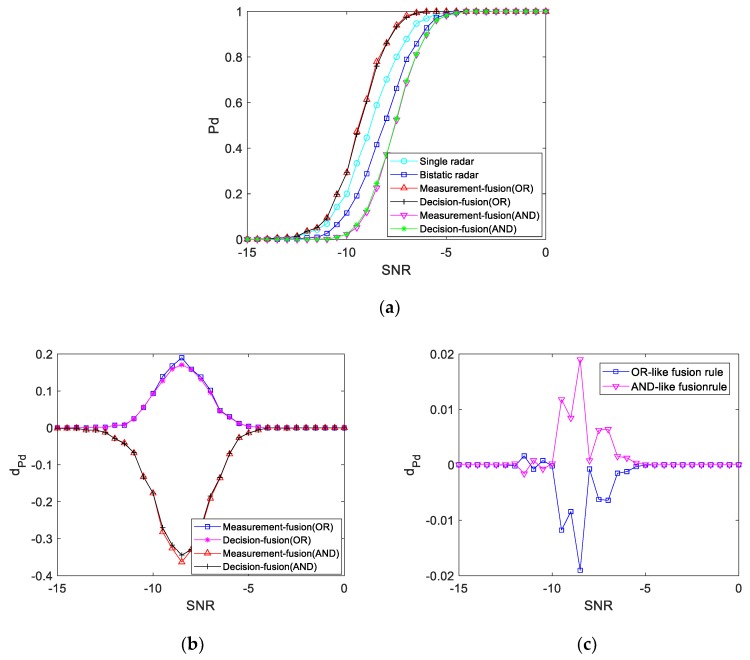
Single radar echo power is greater than bistatic radar echo power: (**a**) Detection performance curve; (**b**) Difference value of the detection performance curve with a single radar; (**c**) Difference value of the fusion detection performance curve.

When the bistatic radar echo power at the target position is greater than the single radar echo power, the detection performance curve obtained by simulation for this is shown in Figure 15. It can be seen from the figure that the detection probability of the three systems is different when the SNR is between −13 and −5 dB. When the SNR is −8 dB, the detection probability of the single radar echo is 70.2%, the detection probability of the double base echo is 53.1%, the fusion detection probability of the OR-like fusion rule is 86.1%, and the fusion detection probability of the AND-like fusion rule is 37.2%. That is, under the same SNR, the detection performance of the single radar echo is better than that of the bistatic radar echo, and the detection probability is higher. After fusion detection of the OR-like fusion rule, the detection performance curve is better than that of the bistatic radar system and single radar system. Additionally, for the AND-like fusion rule, the detection performance curve is worse than that of the bistatic radar system and single radar system.

According to the simulation analysis of the above three different situations, the results are as follows. For the fusion rules, there is a OR-like fusion rule and AND-like fusion rule, where the detection probability under the OR-like fusion rule is high, and the performance of dual sensors is improved comprehensively. The detection probability is low under the AND-like fusion rule, and the dual sensor system becomes a single sensor detection, that is, the “degradation” of the AND-like fusion under the non-uniform SNR. Compared with AND-like fusion, OR-like fusion basically has no degradation problem. The detection condition of one sensor in the dual sensor is very poor, and the detection performance of the dual sensor is better than that of the single sensor. 

In the distributed cooperative detection system, OR-like fusion can make full use of the detection ability of each sensor, reflecting the performance advantage of cooperative detection. In this paper, the cooperative detection system of two platforms was mainly designed. The main consideration is to improve the detection probability of enemy targets as much as possible, making full use of the detection ability of each sensor as much as possible, and the complementary performance of a single radar and bistatic radar in the detection area. Therefore, the detection performance under the OR-like fusion rule was mainly considered in the design.

## 5. Conclusions

In this paper, the dual platform cooperative detection system was taken as the research object. We studied the signal processing and target fusion detection of an airborne cooperative detection system under the clutter condition. First, we constructed a dual platform cooperative detection system geometric model, which can form a single radar and bistatic radar system, respectively, and then signal processing. Clutter suppression was achieved by AMTI. Then, the AMTI, pulse compression, and phase-coherent accumulation were used to suppress the direct wave to suppress the influence of the false target generated by the direct wave during target detection. Finally, the echo received by the two receivers was used for fusion detection to improve the detection performance of the target. The experimental results based on the simulated data demonstrated that: 1) The bistatic radar system with a split transceiver had a larger detection distance than the single radar system, but the influence of the clutter was greater; 2) the direct wave could be eliminated effectively, and no false target could be formed after suppression; 3) the detection probability of the bistatic radar system with split transceivers was higher than that of the single radar system; and 4) the detection probability of signal fusion detection based on two receivers was higher than that of the bistatic radar system and single radar system. The dual platform cooperative detection system constructed in this paper has the advantages of a bistatic radar system and single radar system. The detection range was extended and the detection performance improved.

As for further research approaches, a comparative analysis of different fusion detection methods will be conducted to identify a better fusion detection method. In addition, we will contact the relevant departments to design the actual flight test and verify the effectiveness of the structure and method designed in this manuscript.

## Figures and Tables

**Table 1:** The notation of the symbols.

Notation	Notes
x	Scalar
$\vec{x}$	Vector
X	Matrix
${\left(\cdot \right)}^{-1}$	Inverse of matrix
| ⋅ |	Absolute value
rect(⋅)	Rectangle function

**Table 2:** Total computational complexity.

Fusion CFAR	CA-CFAR	OS-CFAR
Measurement fusion	$O\left(n_{l}\left(2M+MN\right)\right)$	$O\left(n_{l}\left(2M+MN^{2}\right)\right)$
Decision fusion	$O\left(n_{l}\left(3M+2MN\right)\right)$	$O\left(n_{l}\left(3M+2MN^{2}\right)\right)$

**Table 3:** Settings of simulation parameters.

Parameter Description	Parameters	Value
Transmitter coordinates	$\left(x_{t},y_{t},z_{t}\right)$	(0,0,8) km
Receiver coordinates	$\left(x_{r},y_{r},z_{r}\right)$	(80,20,8) km
Target coordinates	(x,y,z)	(110,40,2) km
Transmitter velocity vector	$V_{t}$	(100,10,0) m/s
Receiver velocity vector	$V_{r}$	(100,10,0) m/s
Target velocity vector	$V_{\mathrm{tg}}$	(−100,−50,0) m/s
Transmitting signal carrier frequency	$f_{c}$	1 GHz
Transmission time width	$T_{p}$	30 us
Transmitted signal bandwidth	B	1 MHz
Pulse repetition frequency	$f_{r}$	3000 Hz
Pulse number	M	64
Sampling frequency	$f_{s}$	2 MHz
Signal-to-noise ratio	SNR	15 dB
Signal-to-clutter ratio	SCR	−35 dB
Signal/direct wave power ratio	$K_{2}$	−15 dB
Four-pulse cancellation coefficient of MTI	h	[1 −3 3 −1]
Number of reference units	N	48
Order values of OS-CFAR	k	18
False alarm probability	$p_{f}$	10^−6^
